# Genetic incompatibility of the reproductive partners: an evolutionary perspective on infertility

**DOI:** 10.1093/humrep/deab221

**Published:** 2021-09-28

**Authors:** Jukka Kekäläinen

**Affiliations:** Department of Environmental and Biological Sciences, University of Eastern Finland, Joensuu, Finland

**Keywords:** cryptic female choice, evolution, fertilisation, genetic incompatibility, infertility, mate choice, personalised reproductive medicine, sexual selection, sperm function

## Abstract

In natural fertilisation, the female reproductive tract allows only a strictly selected sperm subpopulation to proceed in the vicinity of an unfertilised oocyte. Female-mediated sperm selection (also known as cryptic female choice (CFC)) is far from a random process, which frequently biases paternity towards particular males over others. Earlier studies have shown that CFC is a ubiquitous phenomenon in the animal kingdom and often promotes assortative fertilisation between genetically compatible mates. Here, I demonstrate that CFC for genetic compatibility likely also occurs in humans and is mediated by a complex network of interacting male and female genes. I also show that the relative contribution of genetic compatibility (i.e. the male–female interaction effect) to reproductive success is generally high and frequently outweighs the effects of individual males and females. Together, these facts indicate that, along with male- and female-dependent pathological factors, reproductive failure can also result from gamete-level incompatibility of the reproductive partners. Therefore, I argue that a deeper understanding of these evolutionary mechanisms of sperm selection can pave the way towards a more inclusive view of infertility and open novel possibilities for the development of more personalised infertility diagnostics and treatments.

## Introduction

Modern ARTs have helped millions of infertile couples to bypass their reproductive challenges. Thus, development of ART is indisputably one of the greatest achievements of medicine. However, despite the demonstrated efficiency of these treatments, the success rate of ART is still far from perfect, and many couples either fail to achieve pregnancy or need several treatment cycles to attain parenthood (Sakkas *et al.*, 2015; De Geyter *et al.*, 2018). Furthermore, diagnosis of infertility is extremely challenging (e.g. Gelbaya *et al.*, 2014; Oehninger and Ombelet, 2019), and in a significant proportion of couples, the reason for infertility remains unexplained (Ray *et al.*, 2012).

According to the current diagnostic practice, infertility is expected to arise from male- and female-dependent pathological factors or a combination of male and female factors (Gardner *et al.*, 2018). However, in addition to male and female pathologies, natural fertilisation success is also heavily dependent on the ability of sperm to traverse the female reproductive tract in the vicinity of an unfertilised oocyte (Fitzpatrick and Lüpold, 2014; Sakkas *et al.*, 2015). Importantly, it has been estimated that in humans, only about 1 out of 1 000 000 sperm are able to enter female oviducts, and only a few of these cells eventually manage to enter the fertilisation site, the ampulla (Eisenbach and Giojalas, 2006). Therefore, natural fertilisation is a highly selective process, in which only very few sperm cells are able to reach the unfertilised oocyte (Holt and Fazeli, 2015; Hanevik *et al.*, 2016). Consequently, the fertilisation success of sperm is dependent on not only the intrinsic quality of the ejaculate (or the pathology of the female reproductive system) but also on the ability of sperm to successfully interact with the female reproductive tract and the oocyte (Fitzpatrick and Lüpold, 2014). In this sense, functionally relevant phenotypic evaluation of ejaculates may be practically impossible in the absence of the selective factors of the female reproductive tract.

Sperm are incapable of fertilising an oocyte immediately after ejaculation and fertilisation competence is achieved only in the female reproductive tract via a series of physiological changes known as sperm capacitation. Capacitated sperm show intense flagella beating (hyperactivation) and directional motility (chemotaxis) towards the chemical factors secreted by unfertilised oocytes. Only capacitated sperm can undergo the acrosome reaction, penetrate the zona pellucida and eventually bind and fertilise an oocyte. All these processes are strongly dependent on the secretions of the female reproductive tract, the oocyte and its surrounding cumulus and granulosa cells. Together, these female-induced biochemical factors allow highly specific sperm selection, possibly even at the level of individual spermatozoa (Holt and Fazeli, 2015). Traditionally, it has been thought that the female-induced sperm selection mechanisms have evolved primarily to eliminate fertilisation-incompetent sperm or to reduce the risk of polyspermy (Fitzpatrick and Lüpold, 2014; Kekäläinen and Evans, 2018). However, in this article, I show that female-mediated sperm selection can also facilitate assortative fusion between genetically compatible gametes. Based on this evidence, I argue that reproductive failure does not necessarily exclusively represent a pathological condition, but can also result from sexual selection (‘mate choice’) at the level of the gametes. Thus, better integration of this evolutionary concept into current infertility diagnostics may provide novel insights into the development of more accurate and personalised infertility diagnostics and treatments.

## Cryptic female choice and gamete-mediated mate choice

**Figure 1.**
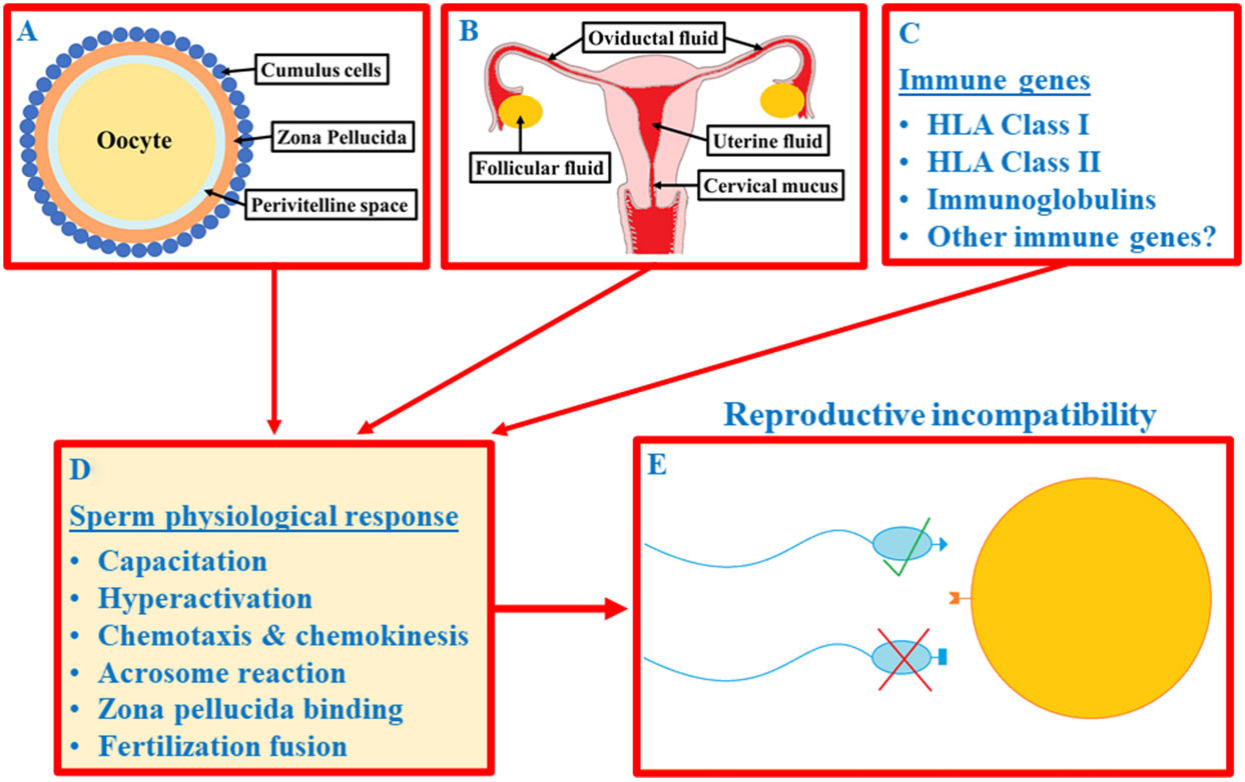
**Schematic illustration of the potential mechanisms of gamete-mediated mate choice in humans.** (**A**) Surface proteins and glycans of the oocyte–cumulus complex and sperm chemoattractants released by these cells (e.g. chemokines, peptides and odourants); (**B**) other female-derived reproductive secretions; (**C**) various genes of the immune system. Together, these female-derived factors cause a number of physiological changes in sperm (**D**) that can selectively bias fertilisation towards the sperm of genetically compatible males (**E**).

Mate choice has traditionally been assumed to occur only at the level of the individuals (i.e. between males and females). However, in many species, it has been demonstrated to continue after mating in the form of cryptic female choice (CFC) (Firman *et al.*, 2017). CFC refers to various female-driven mechanisms that act primarily prior to (or during) fertilisation and bias fertilisation towards the sperm of specific males. In many animal species, CFC is mediated by various female-derived reproductive secretions or via gamete surface molecules, both of which can have major impact on the fertilisation dynamics (Fig.  1). Together, these chemical factors mediate CFC at the level of the gametes (gamete-mediated mate choice (GMMC)) (reviewed by Kekäläinen and Evans, 2018). GMMC has previously been demonstrated to occur primarily in externally fertilising species, such as marine mussels, in which egg-derived sperm chemoattractants selectively change sperm swimming behaviour and thereby promote assortative fertilisation between genetically compatible gametes (e.g. Evans *et al.*, 2012; Oliver and Evans, 2014). Furthermore, several fish studies have demonstrated that a similar fertilisation bias towards particular males can also be mediated by ovarian fluid (Urbach *et al.*, 2005; Dietrich *et al.*, 2008; Rosengrave *et al.*, 2008; Gasparini and Pilastro, 2011; Rosengrave *et al.*, 2016; Geßner *et al.*, 2017).

In internally fertilising species, GMMC occurs within the female reproductive tract, which has hampered experimental attempts to demonstrate GMMC in such species, including humans. These technical difficulties most likely largely explain why experimental evidence of GMMC in humans has been lacking. However, Fitzpatrick *et al.* (2020) recently demonstrated that in humans, follicular fluid highly selectively attracts the sperm of specific males over others, and in this way, facilitates mate choice at the level of the gametes (Fig.  1). Additionally, Jokiniemi *et al.* (2020a,b) demonstrated (also in humans) that sperm performance in different female reproductive secretions (follicular fluid and cervical mucus) is strongly dependent on the male–female combination. In other words, female reproductive secretions were found to selectively increase sperm performance of some males but decrease it in for others. In both studies, sperm performance was also found to be higher in human leucocyte antigen (HLA) dissimilar male–female combinations, which suggests that the female reproductive tract may non-randomly promote gamete fusion between HLA compatible partners. Magris *et al.* (2021) also demonstrated that in addition to HLA, sperm performance in the female reproductive tract is dependent on the structural similarity of male and female immunoglobulins (antibodies). Together with the earlier findings in different animal species, these results indicate that one of the primary functions of GMMC may be to ‘evaluate’ the immunogenetic compatibility of the reproductive partners prior to gamete fusion.

## Genetic interactions, genetic (in)compatibility and reproductive success

The effect of each individual gene on a phenotype is often assumed to be additive, when the combined effect of alleles at two or more gene loci should equal the sum of their separate effects (additive genetic effect). In other words, the phenotypic effect of genes is expected to be independent of all the other genes. However, in all sexually reproducing organisms, the genotypes of the individuals consist of complex networks of genetic interactions (non-additive genetic effects). These interactions can occur among alleles at the same locus, when one allele of a gene masks or overrides the effect of another allele of the same gene (dominance). Additionally, genetic interactions occur between different loci in a phenomenon known as epistasis, in which the phenotypic effect of one locus is enhanced or suppressed by the genotypes at the other locus (or there is a change in the direction of phenotypic effects) (Mackay, 2014). In both dominance and epistasis, the final effect of a gene (or allele) on the phenotype depends on the genotype of the associated genes (or alleles).

Non-additive genetic effects, and especially epistasis, have traditionally been assumed to act as important mechanisms maintaining the reproductive isolation between species (Hart *et al.*, 2018). However, recent studies have demonstrated that epistatic interactions are also common within single species, including humans (Rohlfs *et al.*, 2010; Corbett-Detig *et al.*, 2013; Mackay, 2014; Wang *et al.*, 2017). Importantly, non-additive genetic effects (of the male–female combination) are often much more important determinants of oocyte fertilisation success, embryo survival and fertility traits in general, compared with additive genetic effects of males or females (Palucci *et al.*, 2007; Dziminski *et al.*, 2008; Rodríguez-Muñoz and Tregenza, 2009; Agbali *et al.*, 2010). In other words, reproductive success is frequently more strongly dependent on the male–female compatibility than on individual males and females. This indicates that the same genetic mechanisms responsible for preventing crossbreeding between individuals of different species can also lead to variation in reproductive compatibility between individual males and females within each species. Accordingly, certain male (sperm) genotypes that have high reproductive success with certain female (oocyte) genotypes can have much lower reproductive success with other female genotypes.

## Molecular mechanisms of gamete-mediated mate choice

Widespread evidence for female-mediated fertilisation bias towards the sperm of genetically compatible males suggests that the female reproductive tract and oocytes can identify compatible sperm genotypes based on specific gamete surface molecular markers (Holt and Fazeli, 2015). Consequently, some earlier studies have demonstrated that gamete compatibility genes are expressed on the surface of the sperm and oocytes, and particularly male and female gamete surface proteins have been widely believed to play an important role as molecular targets in compatibility recognition (e.g. Stapper *et al.*, 2015; Springate and Frasier, 2017) (Table  I). Genes coding gamete surface proteins are among the fastest-evolving genes known (Swanson and Vacquier, 2002; Springate and Frasier, 2017), and GMMC is expected to act as an important driver of this evolutionary process, facilitating continual coevolution (reciprocal evolutionary change) between sperm and oocyte proteins (Springate and Frasier, 2017, see below). Due to this coevolutionary process, different variants (alleles) of the coevolving gene pairs often have differential compatibility, which can ultimately lead to complete incompatibility (i.e. reproductive failure) between certain allele pairs (Ziegler *et al.*, 2005).

**Table I:** Oocyte and sperm genes that are known to mediate physical interactions between gametes and are essential for fertilisation in mammals.

Oocyte	Function	Location	Effect	Ref.
*CD9*	Sperm–oocyte fusion	Oocyte surface	Deletion: fertility −40%	1
*CD81*	Sperm–oocyte fusion	Oocyte surface	Deletion: fertility −38%	1
*Juno*	Sperm–oocyte membrane adhesion	Oocyte surface	Deletion: 100% infertility	2
*ZP1-ZP3*	Sperm–oocyte binding/coevolution	Zona pellucida	Sperm–egg compatibility	3,4

**Sperm**				

*Izumo1*	Sperm–oocyte membrane adhesion	Sperm surface after AR	Deletion: 100% infertility	5
*FIMP*	Sperm–oocyte fusion	Sperm equatorial segment	Deletion: severe subfertility	6
*THEM95*	Sperm–oocyte fusion	Sperm plasma membrane	Deletion: 100% infertility	7
*SOF1*	Sperm–oocyte fusion	Sperm plasma membrane	Deletion: 100% infertility	7
*SPACA6*	Sperm–oocyte fusion	Sperm plasma membrane	Deletion: 100% infertility	7
*DCST1/DCST2*	Sperm–oocyte fusion	Sperm plasma membrane	Deletion: 100% infertility	8
*C4BPA (ZP3R)*	Sperm–oocyte binding/coevolution	Sperm plasma membrane	Sperm–egg compatibility	3,4
*PKDREJ*	Zona pellucida (ZP) binding	Sperm acrosome	Mutation: lower fertility	9
*CRISP1/CRISP2*	Sperm–oocyte interaction	Sperm plasma membrane	Blocking: lower fertility	9
*PH-20*	Cumulus penetration + ZP binding	Sperm plasma membrane	Deletion: delayed fertilisation	9,10
*Zonadhesin*	ZP binding	Sperm acrosome	Blocking: lower fertility	9

See also Gahlay and Rajput (2020) for a comprehensive list of sperm genes involved in the interaction of the sperm with the female reproductive tract interaction.

AR, Acrosome reaction.

References: 1. Rubinstein *et al.*, 2006; 2. Bianchi *et al.*, 2014; 3. Rohlfs *et al.*, 2010; 4. Hart *et al.*, 2018; 5. Inoue *et al.*, 2005; 6. Fujihara *et al.*, 2020; 7. Noda *et al.*, 2020; 8. Inoue *et al.*, 2021; 9. Springate and Frasier, 2017; 10. Baba *et al.*, 2002.

In addition to proteins, it has been shown that gamete compatibility is also dependent on their surface carbohydrates (glycans). Ghaderi *et al.* (2011) demonstrated in mice that reproductive incompatibility between males and females is caused by a female immune response against certain (‘mismatched’) sperm surface glycans. Similarly, Kekäläinen and Evans (2017) showed in a marine mussel that egg-derived chemical factors trigger structural changes in sperm surface glycans and sperm fertilisation capability, and that the strength of these physiological changes is strongly dependent on the male-female combination. Kekäläinen and Evans (2017) also demonstrated that the compatibility verification process of the gametes likely commences before the physical contact of the sperm and oocytes, via chemical signals secreted by the female reproductive tract and unfertilised oocytes (reviewed by Kekäläinen and Evans, 2018). This is important, because during the fertilisation process, sperm are exposed to multiple female-derived reproductive secretions, including follicular fluid, oviductal fluid, uterine fluid and cervical mucus, indicating that the selection of genetically compatible sperm can occur in different parts of the female reproductive tract.

## Potential (in)compatibility genes in humans and other mammals

The identities of interacting male and female genes responsible for gamete-level incompatibilities are still largely unclear, and only a few potentially interacting candidate genes have been found. This is largely due to the fact that only one directly interacting sperm–oocyte ‘binding protein’ pair has so far been identified (*Izumo1*–*Juno*: Bianchi *et al.*, 2014; Bianchi and Wright, 2020). However, it is likely that many other gamete surface proteins play important roles as mediators of sperm and oocyte interactions. Supporting this view, gamete surface ‘reproductive’ proteins have been demonstrated to diverge (evolve) rapidly, and continual coevolution between interacting sperm and oocyte proteins is likely a key driver of this divergence (Clark *et al.*, 2009). In this coevolutionary process, one or both ‘members’ of the interacting protein pairs adaptively compensates for changes in the other, which can eventually lead to variation in reproductive compatibility between certain allele pairs of the male and female genes encoding these proteins (Hart *et al.*, 2018).

Accumulating numbers of studies have highlighted that coevolution between sperm and oocyte genes is likely common in mammals, including humans (Vicens and Roldan, 2014; Hart *et al.*, 2018). For example, Grayson (2015) showed that *Izumo1* and *Juno* are coevolving under similar selection pressures, which are at least partly driven by sexual selection. This indicates that some *Izumo1*-*Juno* allele pairs have higher compatibility (gamete fusion success) than others, causing variation in the compatibility between reproductive partners. Rohlfs et al. (2010) also demonstrated in humans that the zona pellucida (glycoprotein layer surrounding the oocyte) gene *ZP3* coevolves with its putative binding partner, *ZP3R*, in sperm (Table  I). Furthermore, recent genome editing studies have revealed several other sperm- and oocyte-specific genes that play an important role in gamete interaction (Abbasi *et al.*, 2020; Fujihara *et al.*, 2020; Lamas-Toranzo *et al.*, 2020; Noda *et al.*, 2020). Although the binding partners of these genes remain to be demonstrated in future studies, all of them have a potential to increase our understanding of the molecular mechanisms of gamete incompatibility.

Many of the key molecules responsible in gamete recognition and binding are not directly situated on gamete surfaces but are dispersed in various female reproductive secretions (Bernabò *et al.*, 2014). Accordingly, sperm behaviour and function in the female reproductive tract are strongly dependent on a large array of female-derived soluble factors, such as chemokines, small peptides and odourant molecules (Brenker *et al.*, 2012). Furthermore, it has been demonstrated that many female-derived factors are transferred from the female reproductive fluids onto the sperm plasma membrane prior to fertilisation (Al-Dossary *et al.*, 2013). For example, two key oocyte surface proteins (CD9 and CD81) known to be involved in sperm–oocyte fusion are also released from oocytes via exosomes (oocyte-derived extracellular vesicles) and interact with sperm before the physical contact of the gametes (Ohnami *et al.*, 2012). Interestingly, many sperm plasma membrane protein genes, such as *SPAM1*, *PMCA4a*, *CRISP1* and *CATSPER*, are also expressed in the female reproductive tract (Griffiths *et al.*, 2008; Al-Dossary *et al.*, 2013; Ernesto *et al.*, 2015; Martinez *et al.*, 2020) and have an important role in regulating sperm function (reviewed by Hernández-Silva and Chirinos, 2019). For example, female-derived CRISP1 proteins were found to regulate sperm Ca^2+^ channels critical for sperm motility (Ernesto *et al.*, 2015). Crucially, fertilisation of the oocytes of *CRISP1* knockout female mice was severely impaired, indicating that female-expressed CRISP1 proteins have a key function in determining the fertilisation capability of sperm.

Together this evidence indicates that genetic compatibility of the reproductive partners may be dependent on the complex network of interacting male and female genes. These genes may not be expressed exclusively on the sperm or oocyte surfaces, but are likely already acting before the physical contact of the gametes via female reproductive tract secretions. This, in turn, indicates that the reproductive compatibility of the partners may be a result of a large number of functionally redundant and possibly relatively weak receptor–ligand interactions (Wright and Bianchi, 2016), which collectively determine the overall compatibility of the partners.

## Clinical significance and future challenges

The primary reason for fertilisation failure in conventional IVF is an unsuccessful sperm–oocyte interaction (Sabetian *et al.*, 2014). It has commonly been assumed that this is primarily caused by some defects in sperm or oocyte membrane proteins mediating the interaction (Sabetian and Shamsir, 2017) or other abnormalities in the ability of sperm to bind and penetrate the zona pellucida (Hamada *et al.*, 2011). However, Firman and Simmon (2015) demonstrated in mice that the success rate of IVF is also dependent on oocyte-driven mechanisms of sperm selection that bias fertilisation towards the sperm of genetically compatible (non-sibling) males. Similarly, Stapper *et al.* (2015) found in sea urchins that eggs non-randomly fused with the sperm that had cell surface protein (bindin) genotypes similar to their own. Finally, Lenz *et al.* (2018) showed in sticklebacks that after controlled IVF, eggs can distinguish sperm genotypes even at the level of individual alleles (haplotypes) and assortatively fuse with complementary sperm haplotypes. Together with the above-mentioned facts, these findings indicate that fertilisation failure does not necessarily represent a pathological condition, but can also result from genetic incompatibility avoidance at the level of the gametes.

Besides affecting the probability of the fertilisation, the compatibility of the gametes at fertilisation has also been demonstrated to be positively associated with embryo survival (Dziminski et al., 2008; Rodríguez-Muñoz and Tregenza, 2009; Agbali *et al.*, 2010; Aguirre *et al.*, 2016; Byrne *et al.*, 2021). Therefore, it is likely that the genetic compatibility of the reproductive partners has a major impact on both fertilisation success and the probability of achieving successful pregnancy and, in this way, influences the overall success rate of infertility treatments. However, according to the definition currently used by the World Health Organization (WHO), infertility is seen as a disease of the reproductive system and is thus assumed to be caused by male- or female-derived pathological factors. In light of previous findings, this may be an overly simplistic view, since it misses the important fact that some male-female (gamete) combinations often ‘match’ better than the others. Therefore, I argue that we need a more inclusive definition of infertility, one which takes into account the possibility that the probability of conception is also affected by the evolutionary mechanisms that strive to ensure the compatibility of the parental genes prior to gamete fusion. This broader definition of infertility can open novel possibilities to better understand the current reliability challenges of infertility diagnostics and to understand why the current diagnostic tests frequently fail to find any clear reason for reproductive failure (Ray *et al.*, 2012) (Table  II).

**Table II:** Potential clinical relevance of investigating genetic incompatibility of the reproductive partners and key challenges for future researchers and clinicians working in the fields of human reproduction and ARTs.

Clinical relevance:
Improved accuracy of infertility diagnostics
Improved predictive value of the semen analyses
More personalised infertility treatments, tailored to each individual couple
Reduced overall costs of ART procedures

**Future research challenges:**

Identify functionally important male and female genes responsible for gamete-level incompatibility
Understand detailed mechanisms of sperm selection in the female reproductive tract
Clarify how the female reproductive tract and oocytes ‘identify’ compatible sperm genotypes
Develop analytical methods for genome-wide characterisation of genetic interactions and genetic interaction networks responsible for gamete-level incompatibility
Investigate the effect of genetic interactions and genetic incompatibility on the health of the offspring

**Future clinical challenges:**

Develop clinical tests for parental genetic compatibility
Develop more realistic functional tests for sperm fertilisation capability and male fertility
Clarify how to prevent the negative impact of genetic incompatibility on reproductive success and offspring health

Fertility traits and reproduction success in general are often dominated by non-additive genetic effects (e.g. Alves *et al.*, 2020). Therefore, in order to predict the probability of conception in individual couples, it is critically important to gain an understanding of how specific male and female genes interact during the fertilisation process. Consequently, deeper understanding of epistatic and dominance interactions between reproductive partners has a great potential to improve the accuracy of infertility diagnostics and facilitate development of more personalised diagnostic tools (Table  II). Personalised reproductive medicine is still in its infancy, and routine clinical tests for parental genetic compatibility are lacking (Beim *et al.*, 2017). However, the rapidly decreasing costs of modern whole-genome sequencing techniques raise an important possibility of including genome-wide characterisation of incompatibility genes in future diagnostics routines. Importantly, recent advances in analytical methods now enable robust identification of genetic interactions and genetic interaction networks from the genome-wide data (e.g. Fang *et al.*, 2019; Sun *et al.*, 2020).

As highlighted above, accumulating evidence indicates that the definitive reproductive incompatibility of the partners is affected by large number of male and female genes, many of which are expressed in female reproductive tract secretions. Consequently, these female-derived secretions could potentially enable diagnosis of the reproductive incompatibility of the partners without the need to fertilise the oocytes (cf. Jokiniemi *et al.*, 2020a,b; Magris *et al.*, 2021). Furthermore, female reproductive secretions could also open novel possibilities for the development of biologically more realistic functional tests for sperm fertilisation capability and male fertility. Thus, besides allowing the evaluation of the reproductive compatibility of the couples, such functional tests could also increase the overall predictive value of semen analyses (Table  II). In practice, sperm functional tests could involve, for example, measuring sperm physiological response to follicular fluid or cervical mucus, both of which can be relatively easily collected during routine ART procedures. Additionally, it has been demonstrated that sperm functional response in ‘non-reproductive’ biological fluids, such as serum, could potentially be used as a reliable indicator of sperm motility and function in female-derived reproductive fluids (Lee *et al.*, 1994; Mandal *et al.*, 2006; Dungdung *et al.*, 2016). This raises an intriguing possibility that the reproductive compatibility of the partners could be screened as a part of the initial infertility testing, which could provide novel opportunities to tailor the following infertility treatments to each couple. However, more studies are needed to experimentally investigate the diagnostic potential of proposed reproductive incompatibility tests and to identify the most suitable candidate genes and other biomarkers to be utilised in such tests.

## Conclusion

According to the current definition, infertility is a disease of the male or female reproductive system. However, an infertility diagnosis can be extremely challenging, and the exact reason for infertility often remains unknown. Recent evolutionary studies have demonstrated that, in addition to being dependent on individual males and females, fertilisation success is also strongly dependent on the reproductive compatibility of the partners (non-additive genetic effects) and that the definitive ‘test’ for male–female compatibility occurs in the female reproductive tract prior to the fertilisation. Therefore, it seems likely that reproductive failure is not exclusively a pathological condition but is also affected by mate choice at the level of the gametes, which reduces the probability of conception between genetically incompatible partners. GMMC is likely based on complex network of interacting male and female genes, which are expressed both on the surface of the gametes and in the female reproductive tract secretions. Besides mediating sperm selection towards those of compatible partners prior to the physical contact of the gametes, female-derived reproductive secretions may also offer novel tools to diagnose the reproductive incompatibility of the partners and thus facilitate development of biologically more realistic fertility tests. Overall, a deeper understanding of molecular basis of reproductive incompatibility may open novel possibilities to overcome the barriers to truly personalised infertility diagnostics and treatments.

## Data availability

No new data were generated or analysed in support of this research.

## Author’s roles

The manuscript was written by the author only.

## Acknowledgements

I would like to thank Annalaura Jokiniemi for comments on the earlier version of the manuscript.

## Funding

Funding was received from the Academy of Finland (308485).

## Conflict of interest

The author declares that no conflicts of interest exist.
